# Neonatal respiratory distress in a reference neonatal unit in Cameroon: an analysis of prevalence, predictors, etiologies and outcomes

**DOI:** 10.11604/pamj.2016.24.152.7066

**Published:** 2016-06-21

**Authors:** Joel Noutakdie Tochie, Simeon-Pierre Choukem, Regina Ndasi Langmia, Esther Barla, Paul Koki-Ndombo

**Affiliations:** 1 Faculty of Health Sciences, University of Buea, Buea, Cameroon; 2 Health and Human Development (2HD) Research Group, Douala, Cameroon; 3 Diabetes and Endocrine Unit, Department of Internal Medicine, Douala General Hospital, Douala, Cameroon; 4 Department of Pediatrics, Muna Memorial Clinic, Douala, Cameroon; 5 Department of Pediatrics, Douala General Hospital, Douala, Cameroon; 6 Mother and Child Centre, Chantal Biya Foundation, Yaoundé, Cameroon; 7 Department of Pediatrics, Faculty of Medicine and Biomedical Sciences, University of Yaoundé 1, Yaoundé, Cameroon

**Keywords:** Neonatal respiratory distress, prevalence, predictors, etiologies, outcomes, Cameroon

## Abstract

**Introduction:**

Neonatal respiratory distress (NRD) is a main cause of neonatal morbidity and mortality in developing countries. Early detection of its risk factors and early treatment of its etiologies are major challenges. However, few studies in developing countries have provided data needed to tackle it. We aimed to determine the prevalence, predictors, etiologies and outcome of NRD in a tertiary health care centre of Cameroon.

**Methods:**

We analyzed the hospital files of all newborns admitted to the Neonatal unit of Douala General Hospital from 1^st^ January 2011 to 28^th^ February 2013. NRD was diagnosed based on the presence of one or more of the following signs: an abnormal respiratory rate, expiratory grunting, nasal flaring, chest wall recessions and thoraco-abdominal asynchrony with or without cyanosis, in their files. Socio-demographic and clinical variables of newborns and their mothers were analyzed using logistic regression analysis.

**Results:**

The prevalence of NRD was 47.5% out of the 703 newborns studied. Acute fetal distress, elective caesarean delivery, APGAR score < 7 at the 1^st^ minute, prematurity, male gender and macrosomia were independent predictors of NRD. The main etiologies were neonatal infections (31%) and transient tachypnea of the newborn (25%). Its neonatal mortality rate was 24.5%, mainly associated with neonatal sepsis and hyaline membrane disease.

**Conclusion:**

NRD is a frequent emergency and causes high morbidity and mortality. Most of its risk factors and etiologies are preventable. Adequate follow-up of pregnancy and labor for timely intervention may improve the neonatal outcomes.

## Introduction

Neonatal respiratory distress (NRD) is clinically identified by the presence of one or more of the following signs: an abnormal respiratory rate or signs of labored breathing, with or without cyanosis [1, 2]. NRD is a frequent neonatal emergency worldwide with reported prevalence rates of 4.24% in Pakistan [3], 18.5% in France [4], 23% in Ivory Coast [5] and 14.5% Burkina Faso [6]. The etiologies and risk factors associated with NRD have not been well cited in low-income countries and particularly sub-Saharan Africa [5–7]. The risk factors found in high-income countries include prematurity, male gender, asphyxia, caesarean delivery, maternal diabetes mellitus, hypertensive disorders of pregnancy, antepartum hemorrhage, multiple pregnancies, and rapid labor [1, 4]. NRD can be caused by a benign etiology, such as transient tachypnea of the newborn, or could be the first manifestation of serious infection, encephalopathy or congenital malformations [8]. Early detection of its risk factors and anticipation of the management of its etiologies are imperative [5, 6, 8]. A tremendous decrease in the NRD specific mortality rate has occurred over the past six decades in high-income countries [7–9] due to several innovations in neonatology that are insufficient or non-existent in low-income countries [5, 6, 10]. However, there have been gradual improvements in health infrastructures and level of care in sub-Saharan Africa over time, yet to the best of our knowledge no well-designed and large-sample study has focused on NRD in the recent years. We aimed to determine the prevalence, predictors, etiologies and outcome of NRD in a referral neonatal unit of Cameroon.

## Methods

### Study design, setting and participants

We analyzed the hospital files of newborns admitted to the Neonatal Unit of Douala General Hospital (DGH) between 1st January 2011 and 28th February 2013 (26 months). As a tertiary hospital, the neonatal unit of DGH is one of the main referral neonatal units of Cameroon. By consecutive convenience sampling, we included all complete and eligible files of newborns admitted within the early neonatal period (first seven days of life). Data were extracted from the files unto a case record form. These data included: **Maternal socio-demographic data:** Maternal age, marital status and employment status; **Obstetric history:** Parity, number of antenatal care visits, multiple pregnancies, diseases during pregnancy, fetal lung maturation when preterm labor < 34weeks, mode of delivery, prolonged rupture of membranes (≥ 12 hours), maternal fever >38°C, meconial stained amniotic fluid, uro-genital tract infections during the last month of pregnancy and fetal distress; **Neonatal variables:** Gestational age, APGAR score at 1st and 5th minutes, gender, birth weight, age on admission, findings on physical examination including the presence/absence of signs of NRD and results of investigations such as full blood count (FBC), C-reactive protein (CRP), blood glucose, X-ray and cardiac ultrasound. The etiologies of NRD in the hospital files were cross-checked to make sure that they were in line with our operational definitions described below. The duration of symptoms and signs of NRD, the duration of hospitalization and the outcome -discharge alive or death- and factors associated with neonatal death were studied for the neonatal outcomes.

### Definition of operational terms and variables

**Neonatal respiratory distress:** The presence of one or more of the following signs: an abnormal respiratory rate (tachypnea > 60 breaths/min, bradypnea < 30 breaths/min, respiratory pauses, or apnea) or signs of labored breathing (expiratory grunting, nasal flaring, intercostal recessions, xyphoid recessions, or thoraco-abdominal asynchrony), with or without cyanosis.

**Non reassuring fetal status or fetal distress:** Fetal heart beat > 160 beats/min or < 120 beats/min and/or meconial stained amniotic fluid with or without an apparent cause of fetal hypoxia.

**Hypoxic ischemic encephalopathy:** An APGAR score ≤ 3 at the 5th minute of life and the presence of neurological signs such as hypotonia, decreased reflexes, coma and/or convulsions. These newborns had an otherwise normal FBC and CRP.

**Neonatal infection:** Any newborn with infectious anamneses (prolonged rupture of membranes, maternal fever around delivery period, maternal uro-genital infections during the last month of pregnancy, etc), clinical signs of infection, and any of the followings: leukocytosis > 25,000/mm3, leucopenia < 5,000/mm3, platelets count < 150,000/mm3, CRP > 20mg/l.

**Transient tachypnea of the newborn (TTN):** Newborns delivered by caesarean section, who have an acute onset of NRD immediately at birth or within some few hours with improvement during the first 24-48 hours of life. These newborns had an otherwise normal physical examination, normal FBC, CRP and blood glucose.

**Meconium aspiration syndrome (MAS):** Onset of NRD immediately at birth or within some few hours, with a history of meconial stained amniotic fluid. These newborns may have hypotonia or decreased reflexes but a normal FBC, CRP and blood glucose.

**Hyaline membrane disease (HMD):** NRD in a preterm newborn, immediately at birth or within the first hours of life with exacerbation during the first 24-48 hours of life, followed by a stable phase till 72 hours, then rapid frank amelioration of NRD between the 3rd and 6th day. These newborns had an otherwise normal physical examination, normal FBC, CRP and blood glucose.

**Congenital heart diseases:** NRD and a heart murmur with echocardiographic confirmation.

**Choanal atresia:** NRD with impossibility of passing a nasogastric tube through the nostrils.

**Congenital diaphragmatic hernia:** NRD with a scaphoid abdomen, distended hemi thorax, displaced cardiac apex beat, and bowel sounds in the chest, with thoracoabdominal X-ray confirmation.

**Esophageal atresia:** NRD with hypersalivation and an impossibility of passing an orogastric tube, with X-ray confirmation.

### Data Analysis

Data was entered in Epi Info 3.5.1 software. Distribution of maternal socio-demographic, obstetrical and neonatal factors associated with NRD were studied and compared between newborns with NRD and those without NRD using the Chi-square test or Fisher exact test where appropriate. Stepwise multiple logistic regression analysis was used to determine the effects of these variables on the development of NRD dichotomized as a yes/no variable. Adjusted odds ratios (OR), their corresponding 95% confidence intervals (95% CI), and p-values were reported. Variables with too much missing data precluding meaningful analyses were excluded. A p-value < 0.05 was considered statistically significant.

### Ethical Considerations

The study was approved by the Institutional Review Board of the Faculty of Health Sciences, University of Buea, Cameroon, and the Medical Director of Douala General Hospital. All files of newborns were consulted in the archive of the Neonatal unit with strict anonymity to ensure confidentiality.

## Results

The files of 703 eligible newborns out of 726 total neonatal admissions (97% response rate) were retained as the study population.

***General characteristics of participants:*** The gestational ages at birth ranged from 24 to 43 weeks (mean: 37.0 ± 3.9 weeks), the majority (66%) being term newborns. Male newborns represented 52.8%. The average birth weight was 2805 ± 870 g (range: 245 to 5310 g). The majority of the newborns (61.3%) were of normal birth weight, 33.1% had low birth weight (<2500g) and 5.6% had macrosomia (≥ 4000g). Most of them were admitted within 0 to 24 hours after birth (Table 1). The mean maternal age was 30.3±5.2 years (range: 16 to 50 years), with 145 (20.6%) mothers aged 35 years or above. The majority of them (91.3%) were married. Regarding the number of antenatal care visits, 533 (75.8%) had respected the recommended four or more, 86 (12.2%) did less than four, while the data were missing in 84 files. The rest of the general characteristics are reported in Table 1.

**Table 1:** General characteristics of the study participants

Variable	Number of newborns N =703	Frequency (%)
**Characteristics of newborns**		
**Gestational Age**		
Preterm (< 37 weeks)	228	32.4
Term (37-41 weeks)	464	66.0
Post term ( ≥ 42 weeks)	11	1.6
APGAR 1^st^ Minute*		
< 7	211	30.0
≥ 7	476	67.7
**Age at admission**		
0-24h	589	83.8
24 -48h	64	9.1
48h < age < 7days	50	7.1
**Maternal and obstetric characteristics**		
**Parity**		
Nulliparity (0)	250	35.6
Primiparity (1)	200	28.4
Multiparity (2-4)	237	33.7
Grand Multiparity (≥5)	16	2.3
**Mode of delivery**		
Vaginal	487	69.3
Elective CS	80	11.4
Emergency CS	136	19.3

* 16 missing; ANC: Antenatal Care; CS: Cesarean Section

**Figure 1:**
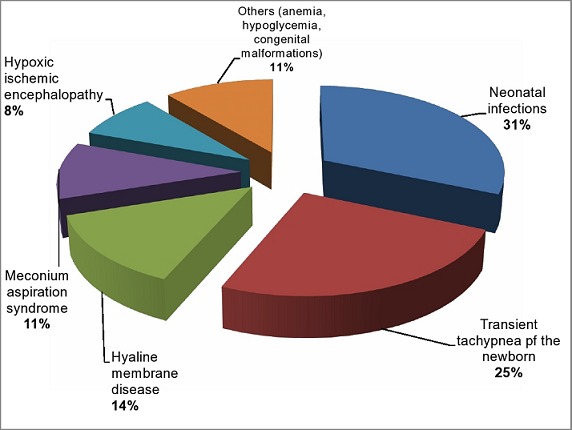
Etiologies of neonatal respiratory distress

***Prevalence, predictors and etiologies of NRD:*** Out of the 703 newborns, 334 had NRD, giving a prevalence of 47.5%. Newborns with NRD and those without NRD had similar mean birth weight (2759±954 vs. 2845±748 respectively), mean maternal age (30.3±5.3 vs. 30.3±5.0 respectively) and maternal parity (1.3±1.8 vs. 1.2±1.7 respectively). In a multiple logistic regression model, prematurity, male gender, high birth weight (≥ 4000 grams), an APGAR score < 7 at the 1st minute, fetal distress and elective caesarean delivery were independent predictors of NRD (Table 2). Attending four or more antenatal visits, prolonged rupture of membranes above 12 hours and intra-partum maternal fever independently reduced the odds of neonatal respiratory distress. None of the maternal factors (maternal age, marital status and parity) was associated with NRD. The etiologies of NRD in the 334 newborns were dominated by neonatal infections (Figure 1).

**Table 2:** Factors independently associated with neonatal respiratory distress

	Adjusted OR	95% CI	p-value
**Neonatal factors**			
Prematurity	1.74	1.20 – 2.95	0.039
Gender Male: Female	1.44	1.04 – 2.01	0.027
High birth weight	2.27	1.06 – 4.87	0.034
APGAR at 1 min < 7	5.19	3.59 – 7.50	< 0.0001
**Obstetrical factors**			
Fetal distress	5.59	3.84 – 8.14	<0.001
Elective cesarean section	3.61	2.01 – 4.08	0.004
Number of antenatal care visits ≥ 4	0.39	0.16 - 0.98	0.045
Prolonged rupture of membranes > 12 hours	0.64	0.44 – 0.93	0.02
Maternal fever > 38°c	0.57	0.33 – 0.99	0.047

**Figure 2:**
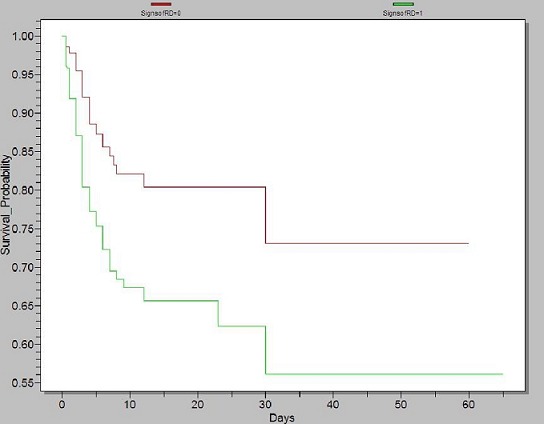
Kaplan-Meier survival comparison between newborns with NRD and those without

*Neonatal Outcome:* eighty-two (24.5%) of the 334 newborns with NRD died compared with 42 (11.4%) of the 369 newborns without NRD (OR = 2.53 [95% CI = 1.69-3.80]; p < 0.01). Seventy four (90.2%) of the NRD deaths occurred within the early neonatal period (0-7 days). Of the 82 deaths, 35 (42.7%) occurred in neonatal sepsis, 20 (24.2%) in hyaline membrane disease, 13 (15.9%) in hypoxic ischemic encephalopathy, 11 (13.4%) in congenital malformations and 3 (3.6%) in meconium aspiration syndrome. Figure 2 compares the survival probability between newborns with NRD and those without NRD using a Kaplan-Meier survival plot. The difference was statistically significant (p < 0.001 for both Log-Rank and Wilcoxon tests). No death was recorded in newborns with TTN, neonatal hypoglycemia and neonatal anemia.

## Discussion

We found a very high prevalence, 47.5% of newborns with respiratory distress. The neonatal unit of DGH is the main referral unit in the city and as such, it concentrates most neonatal emergencies like NRD. While an unpublished study in a Moroccan teaching hospital neonatal unit reported a prevalence of 9.83% in 75 newborn [11], a prevalence of 61.5% was found in neonates admitted in an intensive care unit dedicated to respiratory distress in Istanbul, Turkey [8]. Independent neonatal predictors of NRD were an APGAR score < 7 at the 1st minute, prematurity, male gender and birth weight ≥ 4000 grams. The APGAR score is usually criticized because it does not accurately identify or predict subsequent acute respiratory disorders and neurodevelopmental outcome of the newborn, and many consider it obsolete. However, until a simpler and more useful scoring system for assessing neonates is developed, the APGAR score remains a valid and rapid index for assessing cardiorespiratory adaptation at birth and the effectiveness of resuscitation [12, 13]. This finding is consistent with observations by Chalancon et al. in 2012 [4].

Other authors also reported prematurity as a predictor of NRD [14]. Prematurity goes along with structural and functional immaturity of the lungs (a deficiency in pulmonary surfactant) [15]. The association of NRD with male gender is explained by the antagonism of pulmonary maturation by the higher concentration of androgens [16]. The role of macrosomia as a predictor of NRD can be attributed to the increased incidence of intrapartum fetal distress, shoulder dystocia, instrumental vaginal deliveries with birth injuries, and neoanatal hypoglycemia that is usually associated with macrosomic newborns. Atiye et al., in Turkey [8] and Obama et al. [17], in Cameroon got similar results. Independent obstetrical predictors of NRD were acute fetal distress and elective caesarean delivery. Baby born by elective cesarean delivery lose the beneficial effects (reduction in lung water, enhanced catecholamine levels, secretion of surfactant stores into the alveolar space and pulmonary vasodilatation) conveyed by normal labor [18]. Our observation is in line with that reported in many other studies [19, 20]. Attending four or more antenatal visits (ANC), prolonged rupture of membranes above 12 hours and intrapartum maternal fever reduced the risk of NRD. A minimum of four ANC visits during pregnancy is recommended by WHO [21]. The good follow-up of pregnancy allows early detection and management of potential threats to the mother and newborn. Similarly to other studies [4], we found that prolonged rupture of membranes > 12 hours and maternal fever > 38°C reduced the odds for NRD. The suggested mechanism involves fetal inflammatory syndrome secondary to prolonged rupture of membranes (regardless of chorioamnionitis) which accelerates pulmonary maturation [22, 23]. The fact that 40.9% of women with prolonged rupture of membranes > 12 hours and maternal fever > 38 °C were on antibiotherapy might also explain the reason why the odds of NRD were reduced in these cases. Our findings are therefore not contradictory, because not every neonate born in a context of chorioamnionitis develops a neonatal infection [22, 24]. The risk of NRD due to maternal socio-demographic characteristics is questionable elsewhere, as recently reported [4, 25]. We as well did not find any association between these maternal factors and NRD. This implies that NRD is not determined by pre-existing maternal characteristics; instead, it results from the interaction of acute obstetrical events (like elective cesarean section and acute fetal distress) with neonatal characteristics (prematurity, APGAR score <7 at the 1st minute, macrosomia and male gender).

The main etiologies of NRD were neonatal infections, transient tachypnea of the newborn and hyaline membrane disease and meconium aspiration. As expected, these etiologies of NRD are in phase with risk factors identified in this study. Thus, early diagnosis and treatment of threatened preterm labor might prevent neonatal infections and hyaline membrane disease; meticulously weighing the benefits of normal labor against the risk of NRD following elective caesarean section might prevent transient tachypnea of the newborn; regular fetal monitoring during pregnancy and labor for early detection of fetal distress and timely intervention might prevent meconium aspiration syndrome and hypoxic ischemic encephalopathy. The neonatal fatality rate due to NRD was 24.5%, of which 90.2% occurred within the early neonatal period (0-7 days). The Kaplan Meier plot confirms the poor survival rate of newborn with NRD. Major causes of NRD death were neonatal sepsis, prematurity with hyaline membrane disease and hypoxic ischemic encephalopathy. Despite the fact that our study setting is a referral hospital and is more equipped to face emergencies, a high NRD specific mortality rate of 24.5% was recorded. Thus NRD is a significant cause of neonatal mortality in our milieu. Older African studies noted NRD specific mortality rate of 50% in Burkina Faso [6] and 59.6% in Morocco [11]. Neonatal sepsis was the major contributor to the death rate amongst NRD patients. This confirms the burden of neonatal infections as the first cause of neonatal mortality in developing countries as reported by the World Health Organization [26].

We acknowledge some limitations of our study. As a retrospective analysis conducted in a single and reference center, some data were missing and our results may not be generalized to the entire country. However, based on well documented medical files, a large sample size and a 95% response rate, we have used robust statistical methods to provide novel information on neonatal health.

## Conclusion

Neonatal respiratory distress affects almost half of newborns. It is a major cause of neonatal admissions and has a high mortality rate. Many of its significant risk factors and etiologies are preventable. Adequate follow-up of pregnancy and labor for early detection of risk factors and timely intervention may improve the outcome of neonatal respiratory distress. Overall, our results emphasize the urgent need of improving obstetric care and equipping neonatal care units in order to tackle neonatal respiratory distress.

### What is known about this topic

- Neonatal respiratory distress is a frequent cause of neonatal admission and mortality in low-income countries;
- Low-income countries do not dispose optimal means for its management;
- Prevention of neonatal respiratory distress by anticipating on its predictors and aetiologies is of significant importance for these low-resource countries.

### What this study adds

- Uses a large sample to confirm the high prevalence of neonatal respiratory distress in a low-income country, and identifies its main aetiologies: neonatal infections, transient tachypnoea of the newborn, hyaline membrane disease, meconium aspiration syndrome and hypoxic ischemic encephalopathy;
- Identifies independent predictors of neonatal respiratory distress in sub-Saharan Africa: foetal distress, elective caesarean delivery, APGAR score < 7 at the 1st minute, prematurity, male gender and birth weight ≥ 4000 grams;
- Provides a survival profile of neonatal respiratory distress thus emphasing on the urgent need to improve obstetric care and equip neonatal care units in order to tackle neonatal respiratory distress in Cameroon.
